# Social Phobia Is Associated with Delayed Onset of Chickenpox, Measles, and Mumps Infections

**DOI:** 10.3389/fpsyt.2016.00203

**Published:** 2016-12-27

**Authors:** Vladeta Ajdacic-Gross, Aleksandra Aleksandrowicz, Stephanie Rodgers, Mario Müller, Wolfram Kawohl, Wulf Rössler, Enrique Castelao, Caroline Vandeleur, Roland von Känel, Margot Mutsch, Roselind Lieb, Martin Preisig

**Affiliations:** ^1^Department of Psychiatry, Psychotherapy and Psychosomatics, Psychiatric Hospital, University of Zürich, Zürich, Switzerland; ^2^Epidemiology, Biostatistics and Prevention Institute, University of Zürich, Zürich, Switzerland; ^3^Collegium Helveticum, Swiss Federal Institute of Technology, University of Zürich, Zürich, Switzerland; ^4^Institute of Psychiatry, Laboratory of Neuroscience (LIM27), University of Sao Paulo, Sao Paulo, Brazil; ^5^Department of Psychiatry, University Hospital of Lausanne, Lausanne, Switzerland; ^6^Department of Neurology, Bern University Hospital, Clinic Barmelweid, Barmelweid, Switzerland; ^7^Department of Psychology, Division of Clinical Psychology and Epidemiology, University of Basel, Basel, Switzerland

**Keywords:** social phobia, anxiety disorders, childhood diseases, infectious diseases, epidemiology

## Abstract

**Objective:**

Evidence showing that infectious diseases in childhood play an important role in the etiopathogenesis of neurodevelopmental and other mental disorders is growing. The aim of this study was to explore the timing of common childhood diseases in early-onset anxiety disorders.

**Materials and methods:**

We analyzed data from PsyCoLaus, a large Swiss Population Cohort Study (*N* = 3720). In this study, we regressed overanxious disorder, separation anxiety disorder, social phobia, and specific phobias on the age of onset of several childhood diseases, always adjusting for the other anxiety disorders listed above and for sex.

**Results:**

The timing of viral childhood diseases (chickenpox, measles, and mumps) was consistently delayed in social phobia, notably both in men and women. We found no evidence for a reversed sequence of onset of phobia symptoms before that of the infections included.

**Conclusion:**

Social phobia was the only early anxiety disorder to show an association with a delayed onset of common viral childhood diseases.

## Introduction

Evidence showing that infectious diseases in childhood can play a crucial role in the etiopathogenesis of mental disorders is growing (1). This relation has been a long-standing issue in psychiatric epidemiology, but research activity has recently intensified. Current topics include prenatal infections with *Toxoplasma gondii*, rubella, and other infectious diseases (2, 3) as risk factors for neurodevelopmental disorders and schizophrenia. Furthermore, a series of postnatal infectious agents was reported in association with schizophrenia/psychosis (4–6). The pediatric autoimmune neuropsychiatric disorders associated with streptococcal infections (PANDAS) model also postulates a crucial role for autoantibodies against basal ganglia tissue appearing after infections with group A streptococci in the development of attention deficit hyperactivity disorder, Gilles de la Tourette syndrome, and obsessive–compulsive disorder (OCD) (7, 8). Finally, recent analyses have shed new light on the role of infectious diseases in anxiety disorders (9).

On a formal level, there are four models of how infectious diseases could affect the risk for mental disorders. The models can be illustrated using the associations between Epstein–Barr virus (EBV) and multiple sclerosis (MS) or psychotic disorders. The first model relates to the mere occurrence of an infection. For example, an EBV infection is a necessary precondition for later MS onset: while the seroprevalence for EBV is high, i.e., it covers 90% of the adult population or more (10), almost no one from the negative seroprevalence group develops MS. The second model includes the severity of an infection, for example, due to a dysfunctional immune response and/or due to a combination with genetic or other types of vulnerabilities. The third model considers the timing of childhood infections. Deviations from typical timing, in particular delayed childhood infectious diseases such as chickenpox, measles, mumps, or rubella, are well known to lead to a more severe disease course with more complications (11–14). This also applies to delayed EBV infections, which increase the risk for mononucleosis and probably also for MS (15). Finally, the fourth model focuses on the sequence of childhood infections. This is a possible reason for why infectious disease occurring earlier in life than typically expected might have harmful effects. For example, EBV infections in the first years of life have been linked to psychotic experiences (16). Both the timing and sequence of childhood infections can be assumed to be subjected to evolutionary optimization.

There is a lack of research systematically examining how the timing of infections and other inflammatory insults (17) impacts the risk for mental disorders. This study focused on the timing of childhood diseases with regard to early-onset anxiety disorders (separation anxiety disorder, overanxious disorder, specific phobias, and social phobia). The aim of the study was to examine whether deviations in age of onset of childhood infectious diseases are associated with the early-onset anxiety disorders. We used a two-step approach. First, we examined whether the age of onset of infections differs in cases with and without a specific disorder. The analyses were adjusted for common confounding factors and other early-onset anxiety disorders. In the case of delayed onset, we examined whether the sequence of a childhood infectious disease and the first symptoms of an anxiety disorder might have induced the result.

## Materials and Methods

### The PsyCoLaus Study

The analysis was carried out within the framework of PsyCoLaus, a large epidemiological study conducted in Switzerland. The PsyCoLaus study (18) is the psychiatric part of the population-based CoLaus study (19). The participants in the CoLaus study were randomly selected from the population of the city of Lausanne (Switzerland). The assessment of the subjects took place between 2003 and 2006 in an outpatient clinic (19, 20). It included an interview with a semi-structured questionnaire, as well as the collection of clinical data and blood samples. One year after their initial assessment, CoLaus participants aged between 35 and 66 were asked to participate in the PsyCoLaus study. Subsequently, a total of 3,720 individuals (67%) agreed to participate (18). One major goal of PsyCoLaus was to collect data on the prevalence of psychiatric syndromes/disorders.

A French version of the semi-structured diagnostic interview for genetic studies (DIGS) (21, 22) was used in the PsyCoLaus study to assess a broad spectrum of DSM-IV Axis I criteria. Moreover, the DIGS allowed for gathering additional information about the course and chronology of comorbid features (18). However, the brief phobia section of the DIGS was replaced by the corresponding sections from the Schedule for Affective Disorders and Schizophrenia-Lifetime Version (23).

The study was approved by the Ethics Committee of the University of Lausanne. All participants gave their written informed consent at study enrollment in accordance with the Declaration of Helsinki.

### Childhood Diseases/Disorders and Age of Onset

The early-onset anxiety disorders are characterized by an early age of onset of first symptoms, i.e., mostly before adolescence. While separation anxiety disorder is defined by excessive anxiety of being separated from parents or significant others, overanxious disorder was introduced as a childhood form of general anxiety disorder and in DSM-IV subsumed within this disorder. Specific phobias are a heterogeneous group of disorders (24) related to specific animals, objects, or situations. In social phobia, these are social situations. Typically, girls and women are more frequently affected by early-onset anxiety disorders. The sex ratios can reach a factor of 3 (specific phobias).

The following childhood infections were included in the analyses: pertussis, chickenpox, measles, mumps, rubella, and scarlet fever. The information on infectious diseases and other related conditions was derived using an extended version of the medical history part of the DIGS and was based on self-reporting. In the interview, participants were asked questions about ever having been diagnosed with various infectious diseases, diseases of the nervous system, cardiovascular, respiratory, gastrointestinal, metabolic and dermatological conditions, as well as allergies and hormonal problems. For each disease group, a screening question was asked, followed by more specific questions in the case of an affirmative response. In the section on childhood diseases, chickenpox, measles, and mumps were explicitely asked, whereas information regarding pertussis, rubella, and scarlet fever was extracted from the free text field of this section. These questions routinely included the age of onset of a disease or the first occurrence of symptoms of a mental disorder. Information such as “so early that I cannot remember” or “since I can remember” or age below 2 was replaced by a random value between 2 and 5.

### Statistical Analysis

The analyses followed the conventional analysis design of univariate, bivariate, and multivariate analyses. In bi- and multivariate regression models, the age of onset of infectious diseases was implemented as a predictor of any of the examined early-onset anxiety disorders. In regression analyses, the onset age of infectious diseases was limited to 16 years in order to exclude outliers (14.8% in rubella, and 2.3–6.6% in other infectious diseases). In addition, the age data were smoothed by square root transformation to approach a normal distribution. Each regression analysis relied on a subsample of subjects who reported the infectious disease in question (and not on the whole sample). The analyses were routinely adjusted for:

- sex: in the analysis of overall data;
- other early-onset anxiety disorders (separation anxiety disorder, overanxious disorder, specific phobias, and social phobia) apart from the dependent variable.

Additional adjustments introduced in a one-by-one manner included the education level (three categories), the age of the participants (because of the possibility of recalling a higher age for any events among older age groups), and childhood adversities (fear of parental punishment, parental quarrels, growing up in children’s home).

In the case of statistically significant and consistent results, the analysis design was supplemented by an additional comparison of the observed and expected cases relating to the sequence of a childhood disease and the first symptoms of an early-onset anxiety disorder in order to ascertain that the sequence remained unchanged. Since the relevant information on the age of onset was available only for a subset of subjects (those reporting the respective infectious disease and simultaneously indicating the age of onset of symptoms of an early-onset anxiety disorder), a consistent framework for inference statistics was missing and had to be replaced by a bootstrapping procedure (25). In this procedure, we created for each run a subsample of 1:1 matched controls from the overall sample positively reporting the infectious disease in question. For each run, the hypothetical number of controls was determined in whom an infectious disease occurred before or in the same year as in the corresponding case (relating to the year of onset of first symptoms of an anxiety disorder). We performed 1,000 runs in order to fix the hypothetical sequence and treated it, for the sake of simplicity, as the underlying population sequence. Therefore, the current sequence of infectious diseases and the first symptoms of an early-onset anxiety disorder were tested as the comparison between observed and theoretical frequencies.

Furthermore, in the case of statistically significant and consistent results, we additionally examined the associations between the early-onset anxiety disorders and the frequencies of reported chickenpox, mumps, and measles infections.

The analyses were routinely carried out with the overall data as well as for males and females separately. Moreover, they were repeated for measles after excluding those born in or after 1963 (decreasing endemicity of measles in part due to due to a national vaccination policy). The basic analyses and programming were carried out using SPSS Statistics (version 21). The bootstrapping procedure was programed in a SAS (version 9.3) macro.

## Results

The mean age of onset of first symptoms was 7.0 (SD 3.5) years in overanxious disorder, 5.2 (SD 2.2) years in separation anxiety disorder, 12.1 (SD 9.8) years in specific phobias and 10.9 (SD 8.2) years in social phobia. Table 1 displays the average onset age of common viral and bacterial childhood diseases in early anxiety disorders.

**Table 1:** **Onset age of childhood infectious diseases overall and per anxiety disorder; outliers above 16 years excluded; mean and 95% confidence interval**.

	Chickenpox *N* = 2566	Measles *N* = 2390	Mumps *N* = 1936	Rubella *N* = 201	Pertussis *N* = 256	Scarlet fever *N* = 146
Overall	6.05 (5.95–6.14)	6.06 (5.97–6.16)	7.04 (6.93–7.16)	7.55 (7.01–8.01)	6.39 (6.06–6.72)	7.46 (6.97–7.95)
Separation anxiety disorder	5.76 (5.41–6.12)	5.93 (5.53–6.33)	7.46 (6.90–8.01)	7.11 (5.42–8.80)	7.14 (4.86–9.43)	7.85 (6.07–9.62)
Overanxious disorder	5.98 (5.64–6.33)	6.30 (5.98–6.63)	7.38 (6.93–7.82)	7.89 (6.01–9.78)	6.18 (4.87–7.49)	7.50 (4.47–10.53)
Specific phobia	5.99 (5.76–6.22)	6.16 (5.93–6.39)	7.15 (6.86–7.44)	7.40 (6.36–8.43)	6.39 (5.68–7.10)	6.64 (5.52–7.76)
Social phobia	6.50 (6.22–6.77)	6.67 (6.40–6.93)	7.60 (7.25–7.95)	7.54 (6.07–9.00)	6.83 (5.70–7.96)	8.15 (6.84–9.46)

Table 2 shows the results for social phobia regressed on the age of onset of chickenpox, measles, and mumps for the whole sample and the results for separation anxiety disorders (only males) regressed on the age of onset of mumps. The analyses in other anxiety disorders did not yield significant estimates (results not shown). Neither the adjustment for covariates (other early anxiety disorders and sex) and potential confounders (education level, age of subjects, and childhood adversities) nor the replication of the analyses after excluding subjects born in 1963 or later yielded any noteworthy differences. However, replication by sex-specific analyses showed that in social phobia the onset of chickenpox, measles, and mumps was consistently delayed in both sexes. The odds ratios based on square root smoothed values for age were 1.8 (1.2–2.7), 2.1 (1.3–3.2), and 2.0 (1.3–3.0) in males, and 1.4 (1.0–1.9), 1.7 (1.2–2.2), and 1.4 (1.0–2.0) in females.

**Table 2:** **Regression analysis models with social phobia regressed on age of onset in chickenpox (model 1), measles (model 2), and mumps (model 3); separation anxiety disorder on age of onset in mumps (only males; model 4); age smoothed by square root transformation; odds ratios and 95% confidence interval adjusted in each model for other early anxiety disorders and sex**.

	Model 1 social phobia on chickenpox age	Model 2 social phobia on measles age	Model 3 social phobia on mumps age	Model 4 (males) separation anxiety disorder on mumps age
Age at onset	1.57 (1.23–1.99)	1.76 (1.36–2.28)	1.55 (1.19–2.04)	2.89 (1.55–5.39)
Sex	1.44 (1.11–1.86)	1.39 (1.07–1.82)	1.54 (1.16–2.06)	*
Separation anxiety disorder	1.95 (1.28–2.97)	1.47 (0.93–2.33)	1.46 (0.88–2.42)	*
Overanxious disorder	2.42 (1.69–3.47)	2.47 (1.70–3.59)	2.69 (1.81–3.99)	*
Specific phobia	1.89 (1.31–2.71)	1.79 (1.22–2.63)	2.20 (1.46–3.31)	*

*not applied (see text and Table 3).

The results from analyses addressing the sequence of each childhood disease and the onset of first symptoms of social phobia (or separation anxiety disorder) are displayed in Table 3. In social phobia, the delay in the onset of chickenpox, measles, and mumps did not induce any noteworthy shift between observed and expected cases. However, in separation anxiety disorders, the delay of the onset of mumps was accompanied by an increase of cases with a mumps infection after encountering preliminary separation anxiety symptoms thus indicating that an altered sequence of onsets was involved and induced artificial results.

**Table 3:** **Observed and expected cases with onset of selected childhood disease before or in the same year as the onset of first symptoms of social phobia (overall sample) and separation anxiety disorder (only males)**.

	Social phobia vs. chickenpox	Social phobia vs. measles	Social phobia vs. mumps	Separation anxiety disorder vs. mumps (males)
Subsample *N* total	325	288	247	43
*N* (%) observed	198 (60.9)	185 (64.2)	135 (54.7)	8 (18.6)
*N* (%) expected	208.4 (64.1)	189 (65.7)	140.4 (56.9)	15.3 (35.7)
χ^2^	1.45 (n.s.)	0.27 (n.s.)	0.48 (n.s.)	5.41*

*p < 0.05.

Additional analyses examining the associations between social phobia and reported chickenpox, mumps, and measles infections consistently yielded odds ratios above one, but reached significant levels only regarding mumps (OR = 1.26, CI 1.01–1.57).

## Discussion

This study was the first to examine the timing of childhood diseases in early-onset anxiety disorders (separation anxiety disorder, overanxious disorder, specific phobias, and social phobia). We found that social phobia was associated with a delayed age of viral infectious diseases, in particular with respect to chickenpox, measles, and mumps. Notably, these results were separately replicable in males and females and could not be explained by a reciprocal sequence of reported conditions.

Thus, we offer four possible interpretations of our results. First, the timing of the viral infections could interfere with the changing activity of the immune system, as hypothesized regarding EBV infection in/after adolescence and the EBV–MS link (15, 26). However, social phobia symptoms typically have an early onset before adolescence (27). Since the average delay of chickenpox, measles and mumps infections found in persons with social phobia is rather small (below 1 year), it cannot fill the gap between childhood and adolescence. Therefore, there is no support for a crucial role for adolescence and its concomitants in this disorder.

The second interpretation is more trivial, suggesting that the reporting of chickenpox, measles, and mumps basically relies on the visibility of symptoms such as exanthema. It is noteworthy that these childhood diseases had reached a high seroprevalence of above 90%, before the vaccination campaigns began, thus inducing a ceiling effect in the analyses (12, 14). As mentioned earlier, a higher age at infection is a risk factor for more severe symptoms. This is compatible with the increased frequencies of at least mumps infections reported by subjects with social phobia. With this interpretation, some viral infections are directly associated with the risk for social phobia and mediated by a more severe course and symptoms of the infectious disease.

The third interpretation is closely linked to the second one mentioned earlier. It cannot be excluded that an unknown common factor simultaneously increases the risk for more severe forms of childhood diseases and for social phobia. However, no such factor is currently apparent.

Last but not least, the association between social phobia and delayed age of viral infectious diseases could emerge as an implication of coping with early social phobia symptoms, for example, by avoiding social contact in free time and out-of-school activities. A similar rationale would also apply to separation anxiety and overanxious disorder. However, in these instances, it was not supported by the data.

Thus, the most plausible interpretation for the moment is that viral infections in childhood not only precede but also contribute to the risk for social phobia. The reporting of viral infections in a survey such as PsyCoLaus depends on the perception of manifest symptoms by the subjects. In turn, manifest symptoms are related to a more severe course of these infections and thus also to a stronger involvement of the immune system. Therefore, we hypothesize that the increased risk for social phobia is mediated by a relatively stronger involvement of the immune system. This is in line with models linking infectious diseases with neurodevelopmental disorders—for example, the PANDAS model (8)—or with postinfectious fatigue and depression (28–30). However, in contrast to the PANDAS model, viral—and not, or not only, bacterial—pathogens are involved in social phobia. Moreover, the small delay suggests that apart from the severity of viral childhood diseases a specific age stage, i.e., a specific stage of brain development, should also be considered in social phobia.

While the results of this study show a new facet of how the immune system interferes in the etiopathogenesis of social phobia, they should not be generalized to the whole spectrum of social phobia subtypes. As in other phobias (24) or OCD (31), the analysis of epidemiological parameters, risk factors, and comorbidity patterns split by sex and age at onset of first symptoms indicates some hidden heterogeneity of this disorder, as do clinical studies (32).

### Limitations

As customary in population surveys, the information used was based on self-reporting, including all information on childhood diseases. The participants in PsyCoLaus were adults up to 66 years of age. Therefore, the analyses might be biased by telescoping effects such as those found typically regarding onset of substance use (27, 28) and display an age-dependent recall-bias regarding symptoms and diseases occurring in childhood and youth. However, no evidence for this was found in the analyses adjusted for age.

The diagnosis of social phobias and other disorders was based on information from epidemiological instruments and not on clinical assessment. In some instances, the analyses might also have failed to reveal significant estimates due to small frequencies of cases. While some infectious diseases were directly documented in the DIGS, others were covered by a more general question. Thus, the recall bias might interfere differently, depending on the question format.

The interpretation is solely based on association analyses and results from regression analyses. Therefore, it includes a strong theoretical or speculative component and should be adopted with caution. Ongoing studies covering other infectious diseases and other mental disorders will facilitate a more precise and comprehensive interpretation of the specific link between viral childhood diseases and social phobia.

## Conclusion

The analysis of the timing of childhood diseases in early anxiety disorders has shed new light on the connection between viral childhood diseases—chickenpox, measles, and mumps—and social phobia. While the clinical implications of this study are minor, its theoretical implications are challenging. The results suggest a role for the immune system in impacting the development of early onset anxiety disorders.

## Author Contributions

MP, EC, and CV designed the PsyCoLaus study and acquired the data. VA-G, AA, SR, and MM carried out the analysis. WK, WR, RK, MargM, and RL discussed the preliminary results. VA-G and AA wrote the paper. All the authors contributed to the interpretation of the results and to the critical revision of the manuscript.

## Conflict of Interest Statement

The authors declare that the research was conducted in the absence of any commercial or financial relationships that could be construed as a potential conflict of interest.

## References

[1] Hornig M. The role of microbes and autoimmunity in the pathogenesis of neuropsychiatric illness. Curr Opin Rheumatol (2013) 25:488–795. 10.1097/BOR.0b013e32836208de 23656715

[2] Nickerson JP Richner B Santy K Lequin MH Poretti A Filippi CG Neuroimaging of pediatric intracranial infection – part 2: TORCH, viral, fungal, and parasitic infections. J Neuroimaging (2012) 22:e52–63. 10.1111/j.1552-6569.2011.00700.x 22309611

[3] Adams Waldorf KM McAdams RM. Influence of infection during pregnancy on fetal development. Reproduction (2013) 146:R151–62. 10.1530/REP-13-0232 23884862 PMC4060827

[4] Sperner-Unterweger B. [Biological hypotheses of schizophrenia: possible influences of immunology and endocrinology]. Fortschr Neurol Psychiatr (2005) 73(Suppl 1):S38–43. 10.1055/s-2005-915544 16270243

[5] Yolken RH Torrey EF. Are some cases of psychosis caused by microbial agents? A review of the evidence. Mol Psychiatry (2008) 13:470–9. 10.1038/mp.2008.5 18268502

[6] Torrey EF Bartko JJ Yolken RH. *Toxoplasma gondii* and other risk factors for schizophrenia: an update. Schizophr Bull (2012) 38:642–7. 10.1093/schbul/sbs043 22446566 PMC3329973

[7] Martino D Defazio G Giovannoni G. The PANDAS subgroup of tic disorders and childhood-onset obsessive-compulsive disorder. J Psychosom Res (2009) 67:547–57. 10.1016/j.jpsychores.2009.07.004 19913659

[8] Murphy TK Storch EA Lewin AB Edge PJ Goodman WK Clinical factors associated with pediatric autoimmune neuropsychiatric disorders associated with streptococcal infections. J Pediatr (2012) 160:314–9. 10.1016/j.jpeds.2011.07.012 21868033 PMC3227761

[9] Witthauer C Gloster AT Meyer AH Goodwin RD Lieb R. Comorbidity of infectious diseases and anxiety disorders in adults and its association with quality of life: a community study. Front Public Health (2014) 2:80. 10.3389/fpubh.2014.00080 25072049 PMC4095564

[10] Ruprecht K. [Multiple sclerosis and Epstein-Barr virus: new developments and perspectives]. Nervenarzt (2008) 79:399–407. 10.1007/s00115-007-2335-8 17713752

[11] Wittek M Doerr HW Allwinn R. [Varicella and herpes zoster. Part 1: virology, epidemiology, clinical picture, laboratory diagnostics]. Med Klin (Munich) (2010) 105:334–8. 10.1007/s00063-010-1061-3 20503007

[12] Perry RT Halsey NA. The clinical significance of measles: a review. J Infect Dis (2004) 189(Suppl 1):S4–16. 10.1086/377712 15106083

[13] Stock I [Rubella (German measles) – still a major infectious disease]. Med Monatsschr Pharm (2012) 35:14–22. 22332308

[14] Hviid A Rubin S Muhlemann K. Mumps. Lancet (2008) 371:932–44. 10.1016/S0140-6736(08)60419-5 18342688

[15] Thacker EL Mirzaei F Ascherio A. Infectious mononucleosis and risk for multiple sclerosis: a meta-analysis. Ann Neurol (2006) 59:499–503. 10.1002/ana.20820 16502434

[16] Khandaker GM Stochl J Zammit S Lewis G Jones PB. Childhood Epstein-Barr virus infection and subsequent risk of psychotic experiences in adolescence: a population-based prospective serological study. Schizophr Res (2014) 158:19–24. 10.1016/j.schres.2014.05.019 25048425 PMC4561501

[17] Du Preez A Leveson J Zunszain PA Pariante CM. Inflammatory insults and mental health consequences: does timing matter when it comes to depression? Psychol Med (2016) 46:2041–57. 10.1017/S0033291716000672 27181594 PMC4937234

[18] Preisig M Waeber G Vollenweider P Bovet P Rothen S Vandeleur C The PsyCoLaus study: methodology and characteristics of the sample of a population-based survey on psychiatric disorders and their association with genetic and cardiovascular risk factors. BMC Psychiatry (2009) 9:9. 10.1186/1471-244X-9-9 19292899 PMC2667506

[19] Firmann M Mayor V Vidal PM Bochud M Pecoud A Hayoz D The CoLaus study: a population-based study to investigate the epidemiology and genetic determinants of cardiovascular risk factors and metabolic syndrome. BMC Cardiovasc Disord (2008) 8:6. 10.1186/1471-2261-8-6 18366642 PMC2311269

[20] Novy J Castelao E Preisig M Vidal PM Waeber G Vollenweider P Psychiatric co-morbidities and cardiovascular risk factors in people with lifetime history of epilepsy of an urban community. Clin Neurol Neurosurg (2012) 114:26–30. 10.1016/j.clineuro.2011.08.019 21955581

[21] Nurnberger JI Jr Blehar MC Kaufmann CA York-Cooler C Simpson SG Harkavy-Friedman J Diagnostic interview for genetic studies. Rationale, unique features, and training. NIMH genetics initiative. Arch Gen Psychiatry (1994) 51:849–59; discussion 63–4. 10.1001/archpsyc.1994.03950110009002 7944874

[22] Preisig M Fenton BT Matthey ML Berney A Ferrero F. Diagnostic interview for genetic studies (DIGS): inter-rater and test-retest reliability of the French version. Eur Arch Psychiatry Clin Neurosci (1999) 249:174–9. 10.1007/s004060050084 10449592

[23] Endicott J Spitzer RL A diagnostic interview: the schedule for affective disorders and schizophrenia. Arch Gen Psychiatry (1978) 35:837–44. 10.1001/archpsyc.1978.01770310043002 678037

[24] Ajdacic-Gross V Rodgers S Muller M Hengartner MP Aleksandrowicz A Kawohl W Pure animal phobia is more specific than other specific phobias: epidemiological evidence from the Zurich study, the ZInEP and the PsyCoLaus. Eur Arch Psychiatry Clin Neurosci (2016) 266(6):567–77. 10.1007/s00406-016-0687-4 27001383

[25] Chernick MR Bootstrap Methods: A Guide for Practitioners and Researchers. Hoboken: John Wiley & Sons (2007).

[26] Ascherio A Munger KL. Epstein-Barr virus infection and multiple sclerosis: a review. J Neuroimmune Pharmacol (2010) 5:271–7. 10.1007/s11481-010-9201-3 20369303

[27] Stein MB Stein DJ. Social anxiety disorder. Lancet (2008) 371:1115–25. 10.1016/S0140-6736(08)60488-2 18374843

[28] Anders S Tanaka M Kinney DK. Depression as an evolutionary strategy for defense against infection. Brain Behav Immun (2013) 31:9–22. 10.1016/j.bbi.2012.12.002 23261774

[29] Katz BZ Jason LA. Chronic fatigue syndrome following infections in adolescents. Curr Opin Pediatr (2013) 25:95–102. 10.1097/MOP.0b013e32835c1108 23263024

[30] Dantzer R Heijnen CJ Kavelaars A Laye S Capuron L. The neuroimmune basis of fatigue. Trends Neurosci (2014) 37:39–46. 10.1016/j.tins.2013.10.003 24239063 PMC3889707

[31] Rodgers S Ajdacic-Gross V Kawohl W Muller M Rossler W Hengartner MP Comparing two basic subtypes in OCD across three large community samples: a pure compulsive versus a mixed obsessive-compulsive subtype. Eur Arch Psychiatry Clin Neurosci (2015) 265(8):719–34. 10.1007/s00406-015-0594-0 25827623

[32] Knappe S Beesdo-Baum K Fehm L Stein MB Lieb R Wittchen HU. Social fear and social phobia types among community youth: differential clinical features and vulnerability factors. J Psychiatr Res (2011) 45:111–20. 10.1016/j.jpsychires.2010.05.002 20684833

